# Characterization of patients admitted to specialized geriatric acute care hospital units with the German version of the Standardized Evaluation and Intervention for Seniors at Risk (SEISAR) screening-instrument: a cross-sectional study

**DOI:** 10.1186/s12877-023-04338-7

**Published:** 2023-09-30

**Authors:** Rainer Wirth, J. Verdon, H. Frohnhofen, M. Djukic, M. Meisel, M. Musolf, A. Zinke, H. J. Heppner, M. Jamour, M. Denkinger, U. S. Trampisch

**Affiliations:** 1 Department of Geriatric Medicine, Marien Hospital Herne, Ruhr-Universität Bochum, Herne, Germany; 2 McGill University Health Center, Royal Victoria Hospital, Montreal, QC Canada; 3 Department of Orthopedics and Traumasurgery, Heinrich Heine University, Düsseldorf, Germany; 4 Department of Health, University Witten-Herdecke, Witten, Germany; 5 Department of Geriatric Medicine, Evangelisches Krankenhaus Göttingen-Weende, Göttingen, Germany; 6 MEDICLIN Heart-Center, Coswig, Germany; 7 Department of Geriatric Medicine, Ev. Amalie Sieveking-Krankenhaus, Hamburg, Germany; 8 Department of Geriatric Medicine, St. Marien- und St. Annastiftskrankenhaus, Ludwigshafen, Germany; 9 Department of Geriatric Medicine, Klinikum Bayreuth, Bayreuth, Germany; 10 Department of Internal and Geriatric Medicine, Alb-Donau-Klinikum, Ehingen, Germany; 11 Geriatric Centre Ulm, Agaplesion Bethesda Clinic, Ulm University, Ulm, Germany

**Keywords:** Geriatric assessment, Screening, Allocation, Rehabilitation

## Abstract

**Background:**

The **S**tandardized **E**valuation and **I**ntervention for **S**eniors **a**t **R**isk (SEISAR) screening tool records major geriatric problems, originally applied in the emergency department. Particularly, the distinction of compensated and uncompensated problems is an interesting and new approach. Therefore, we translated the SEISAR in German language and used it to characterize patients in specialized geriatric hospital wards in Germany and to gather initial experience regarding its usability and practicability.

**Methods:**

The tool was translated by three independent specialists in geriatric medicine and backtranslated for quality-assurance by a non-medical English native speaker. In a second step, 8 acute care geriatric hospital departments used the translated version to characterize all consecutive patients admitted over a period of one month between December 2019 and May 2020 at time of admission.

**Results:**

Most of the 756 patients (78%) lived in an own apartment or house prior to hospital admission. Participants had on average 4 compensated and 6 uncompensated problems, a Barthel-Index of 40 pts. on admission with a median increase of 15 points during hospital stay, and a median length of stay of 16 days in the geriatric hospital department.

**Conclusion:**

SEISAR is an interesting standardized brief comprehensive geriatric assessment tool for the identification of compensated and uncompensated health problems in older persons. The data of this study highlights the number, variability, and complexity of geriatric problems in patients treated in specialized acute care geriatric hospital wards in Germany.

**Trial registration:**

German Clinical trial register (DRKS-ID: DRKS00031354 on 27.02.2023).

**Supplementary Information:**

The online version contains supplementary material available at 10.1186/s12877-023-04338-7.

## Background

Geriatrics as a medical specialty developed concomitant to the increasing human lifespan in order to optimize the medical treatment of older multimorbid patients with special regards to functionality and autonomy. The effectiveness of the geriatric concept, including geriatric assessment, multi-professional teamwork and training interventions as main components, has been proven in many studies [1, 2]. However, due to longer lengths of hospital stay and increased personnel requirements, the specialized geriatric treatment generally consumes more immediate health care resources compared to non-geriatric standard treatment. Therefore, specialized geriatric treatment should be applied in patients with increased needs and where it is supposed to be effective.

The **I**dentification of **S**eniors **a**t **R**isk-tool (ISAR) in its original and revised version (ISAR-R) is suggested to identify older patients with increased health care needs. It is a rapid screening tool able to identify seniors at high risk for adverse events after an emergency department (ER) visit [3–5]. However, ISAR identifies a high proportion of older patients at a high risk of adverse events and its usability for the allocation to specialized geriatric hospital wards seems questionable for the current German health care system. In one study in general internal medicine in Germany 58% of patients ≥ 75 years had a positive ISAR screening [6]. In another study from German emergency department patients 80% of patients > 70 years demonstrated a positive ISAR screening [7]. Therefore, the use of ISAR outside of the emergency department seams inadequate for the allocation of patients to a specialized geriatric ward, since it is not sufficiently selective.

To the best of our knowledge, there are currently no effective operationalized criteria for the adequate allocation of older patients to a specialized geriatric hospital ward. Thus, it is still a matter of individual geriatric expertise to perform this task.

Therefore, we searched for objective instruments that potentially could be capable of this task. In this context we came across the **S**tandardized **E**valuation and **I**ntervention for **S**eniors **a**t **R**isk (SEISAR) screening tool [8]. The SEISAR-tool identifies all relevant deficits that are common in the geriatric population and classifies them as “no, problem absent”, problem “present, but compensated” or problem “present, but uncompensated”. Particularly, the differentiation of compensated and uncompensated medical problems is a new approach which seems practical for the desired task. The SEISAR instrument was originally designed to document and characterize the special need for further geriatric outpatient care in patients with positive ISAR-screening discharged home from an emergency department. Intervention on uncompensated problems identified were to occur in the ER or were to be addressed in outpatient follow-up, according to a standardized approach, all in the spirit of optimizing patient safety at and after discharge. However, patients with uncompensated problems were not intended to be admitted to a geriatric hospital unit in the original SEISAR approach. Conversely, we sought to characterize older hospital patients in need of specialized geriatric hospital care with the SEISAR-tool and to gather initial experience regarding its usability and practicability, in an attempt to see if this tool could help to identify those to admit in the future. In addition, we examined how the presence of these geriatric problems could affect functional recovery.

## Methods

The SEISAR screening tool captures 22 deficits in ten domains. The domains comprise communication, cognition, nutrition, mobility, activities of daily living (ADL), medication, behavior and affect, active medical issues, pain management and social life. Additional information is obtained about the source of information and the living arrangements.

In a first step, the original English version of the SEISAR was translated into German language by three independent specialists in geriatric medicine. After harmonization of the translated version, it was backtranslated by a non-medical English native speaker. The result revealed no relevant discrepancies to the original version.

In a second step, eight acute care geriatric hospital departments used the German version of the SEISAR in the hospital routine and completed it in an anonymous manner, i.e. without identifying patient data, for all consecutively admitted patients at time of admission in a period of one month between December 2019 and May 2020. The SEISAR-manual (version 3.1) was provided to all participating departments. The SEISAR assessment was performed on the day of admission by the attending physician under supervision of the respective chief geriatrician in parallel to the routine geriatric assessment, which is generally performed by nursing staff and physiotherapists. Nursing staff and physiotherapists did not see and recognize the results of the SEISAR assessment, but were not actively blinded.

According to the manual an uncompensated problem was defined as being either new or not under control, when no appropriate coping mechanism or strategy has been put into place.

Additionally, it was recorded from which setting the patients were admitted, the length of hospital stay, if an early rehabilitation procedure had been performed and the Barthel-Index (BI) on admission and at discharge. The German version of the BI has a range from 0 to 100 points, with 100 points indicating complete independence in ADL [9]. Early rehabilitation is a formalized procedure in the German health care system with a predefined minimal duration, content of assessments and therapeutic sessions that can be coded and thus lead to the reimbursement of health care costs for the multi-professional geriatric treatment.

### Statistics and ethics

The statistical analysis was performed using SPSS statistical software (SPSS Statistics for Windows, IBM Corp, Version 29, Armonk, NY, USA). Continuous variables are expressed by means, standard deviations (SDs), minimum (min) und maximum (max), or median and interquartile ranges (IQR). Categorical variables are shown as absolute numbers and percentages (n, %). Besides the description of problem prevalence and the number of problems per person, a problem burden score was calculated for each subject (0 = problem absent, 1 = compensated problem, 2 = uncompensated problem, maximal value 44 pts).

For group comparisons, the difference of BI admission – discharge during hospital stay was calculated and categorized as decline or no change versus increase of ≥ 5 pts. We analyzed differences in frequency of problems between the two groups by unpaired t tests. For items with ≥ 15% differences in uncompensated problems, we performed chi square tests. Kruskal-Wallis test was performed for BI and change in BI and type of admission (direct admission from general practitioner of emergency admission vs. transfer from other hospital or other department). Statistical significance is set at *p* < 0.05. The study protocol was approved by the ethical committee of the University of Bochum (Nr. 20-6916-BR).

## Results

We obtained data of 756 patients admitted to eight different geriatric hospital departments in six federal states of Germany. More than half of patients lived alone (56%), one third (34%) lived with spouse or family, and the majority of patients lived in an own apartment or house (78%) prior to hospital admission. The patients were directly transferred from other hospital departments in 64%, directly admitted from the emergency department in 19%, and were referred by the general practitioner in 15%. In 2%, this information was not available. The median length of stay was 16 days (IQR 14–20). For more details see Table 1.

**Fig. 1:**
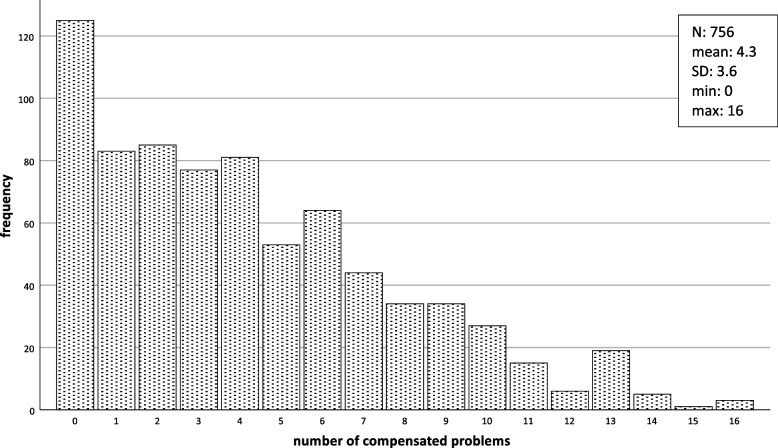
Frequency of compensated problems per patient (*n* = 756)

**Fig. 2:**
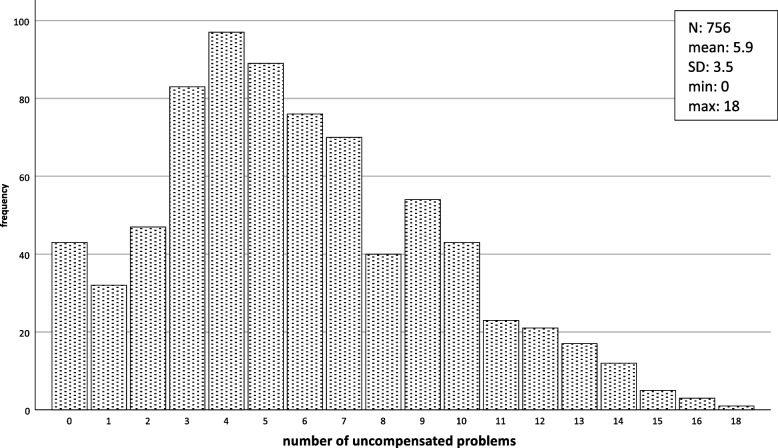
Frequency of uncompensated problems per patient (*n* = 756)

In total, 3,230 problems “present, but compensated” and 4,425 “present, but uncompensated” were reported, i.e. a mean of 4.3 (± 3.6) compensated problems and 5.9 (± 3.5) uncompensated problems per person. Although in 43 subjects (5.7%) no present problem was recorded, 90% of the patients demonstrated between 1.7 and 18.0 uncompensated problems (Figs. 1 and 2).

**Table 1:** Characteristics of the study population (*n* = 756)

	Frequency (%)
Living situation	
Alone	422 (55.8)
With spouse or family	260 (34.4)
Residence with service	29 (3.8)
Unknown	45 (6.0)
Residential status	
House, apartment	593 (78.4)
Nursing home, group home	64 (8.5)
Residence without service	28 (2.4)
Unknown	81 (10.7)
Admission from	
Admission from emergency department	145 (19.2)
Referral from general practitioner	111 (14.7)
Transfer from other hospital department	480 (63.5)
Unknown	20 (2.6)
Duration of early rehabilitation procedure	
< 7 days	55 (7.3)
7–13 days	110 (14.6)
14–21 days	441 (58.3)
> 21 days	149 (19.7)
Unknown	1 (0.1)
	median (IQR)
Length of hospital stay (d)	16 (14–20)
Barthel-Index on admission (pts.)	40 (30–55)
Barthel-Index at discharge (pts.)	60 (45–75)
Increase of Barthel-index (pts.)	15 (5–30)
	mean + SD
Absent problems per patient	11.6 ± 4.0
Compensated problems per patient	4.3 ± 3.6
Uncompensated problems per patient	5.9 ± 3.5
SEISAR problem burden score (missing *n* = 106)	16.1 ± 6.1

*SD *Standard deviation, *IQR *Interquartile ranges

**Fig. 3:**
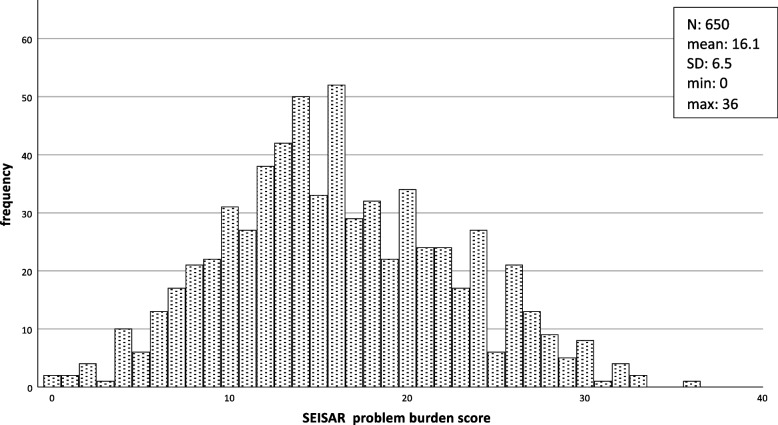
SEISAR problem burden score (number of compensated (1 pt.) + number of uncompensated (2 pts.) problems. The problem burden score could only be calculated in *n* = 650 participants without any missing values

The most prevalent uncompensated problems reported were polypharmacy (73%), falls (60%), persistent presenting symptoms (51%), problems walking (49%) and active comorbidities (46%). The most prevalent compensated problems were impaired vision (54%), impaired hearing (36%), limitations with basic hygiene (33%), difficulties with meal preparation (32%) and problems walking (29%) (Table 2). The mean value of the SEISAR problem burden score was 16.1 (±6.1, min:0, max:36) (Fig. 3).

In 730 patients with data on BI on admission and at discharge (missing data in *n* = 26) the median increase of BI during hospital stay was 15 points. In 46 patients (6.3%) the BI decreased during hospital stay, in 85 subjects (11.6%) BI remained unchanged and 67 patients (9.2%) displayed an increase of 5 pts, whereas the majority of 532 patients (72.9%) demonstrated an increase of BI of ≥ 5 pts during hospital stay.

A group comparison revealed significant differences between patient groups with and without an increase of BI. Patients with an increase in BI during geriatric hospital treatment had a marginally non-significant lower number of uncompensated problems (5.6 vs. 7.2; *p* = 0.06) than those with no change or decrease in BI. The number of compensated problems did not significantly differ between both groups (4.2 vs. 4.3; *p* = 0.73).

However, the problem burden score was significantly different between both groups. Those with an increase in BI had a lower problem burden (15.5 ± 6.3 pts.) than those without increase of BI (19.0 ± 6.5 pts.; *p* < 0.001).

The items “acute confusion/disorientation”, “undiagnosed cognitive problems”, “problems walking/difficulty in using walking aid” and “active co-morbidities” showed differences in between groups of > 15% (Additional file 1). Chi Square tests showed significant differences with a lower prevalence of the above-mentioned uncompensated problems in the group with an increase of BI, respectively.

**Table 2:** Prevalence of problems absent, compensated or uncompensated (*n* = 756*)

	all (*n* = 756)
**Communication:**	
Impaired vision	
absent	267 (35.8)
compensated	402 (54.0)
uncompensated	76 (10.2)
Impaired hearing	
absent	374 (50.7)
compensated	263 (35.6)
uncompensated	101 (13.7)
**Cognition:**	
Acute confusion/disorientation	
absent	496 (66.0)
compensated	60 (8.0)
uncompensated	195 (26.0)
Undiagnosed cognitive problems	
absent	576 (77.2)
compensated	44 (5.9)
uncompensated	126 (16.9)
**Nutrition:**	
Recent weight loss/malnutrition	
absent	431 (57.1)
compensated	58 (7.7)
uncompensated	266 (35.2)
Substance abuse	
absent	708 (94.7)
compensated	9 (1.2)
uncompensated	31 (4.1)
**Mobility:**	
Falls	
absent	223 (29.6)
compensated	81 (10.8)
uncompensated	449 (59.6)
Problems walking/difficulty in using walking aid	
absent	164 (21.9)
compensated	216 (28.8)
uncompensated	369 (49.3)
**Activities of daily living:**	
Difficulties with meal preparation	
absent	303 (40.4)
compensated	242 (32.3)
uncompensated	205 (27.3)
Limitations with basic hygiene	
absent	296 (39.7)
compensated	247 (33.2)
uncompensated	202 (27.1)
Incontinence	
absent	403 (53.9)
compensated	184 (24.6)
uncompensated	161 (21.5)
**Medication:**	
Polypharmacy/new medication	
absent	112 (14.9)
compensated	91 (12.1)
uncompensated	548 (73.0)
Prescription management difficulties	
absent	456 (61.5)
compensated	149 (20.1)
uncompensated	136 (18.4)
**Behavior/affect:**	
Depression	
absent	627 (83.3)
compensated	45 (6.0)
uncompensated	81 (10.7)
Agitation	
absent	690 (92.1)
compensated	21 (2.8)
uncompensated	38 (5.1)
**Active medical issues:**	
Persistent presenting symptoms	
absent	194 (25.8)
compensated	175 (23.3)
uncompensated	382 (50.9)
Active co-morbidities	
absent	237 (31.6)
compensated	171 (22.8)
uncompensated	341 (45.5)
**Pain management:**	
Persistent pain	
absent	343 (45.5.)
compensated	186 (24.7)
uncompensated	225 (29.8)
Joint/bone pain	
absent	366 (48.7)
compensated	177 (23.5)
uncompensated	209 (27.8)
**Social:**	
Insufficient support, lives alone	
absent	330 (44.2)
compensated	216 (29.0)
uncompensated	200 (26.8)
Social isolation/neglect	
absent	567 (76.3)
compensated	128 (17.2)
uncompensated	48 (6.5)
Previously refused service	
absent	640 (86.4)
compensated	65 (8.8)
uncompensated	36 (4.8)

*missing values in up to 2.7% of cases per group

Group comparison with regards to admission type did not reveal significant differences between groups in terms of BI and gain in BI.

## Discussion

The current analysis documents the variety and high variability of compensated and uncompensated functional problems and health-related problems of patients admitted to an acute care geriatric hospital unit in Germany. The SEISAR is a standardized brief comprehensive geriatric assessment, allowing to better overlook the complexity of the patients admitted. Leading uncompensated problems were polypharmacy, falls, walking difficulties, persistent symptoms and active comorbidities. Most prevalent compensated problems were impaired vision, impaired hearing, difficulties with basic hygiene and meal preparation and problems walking. Other typical problems such as acute confusion (uncompensated in 26%) and malnutrition (uncompensated in 35%) were less frequent but still highly prevalent, reflecting the prevalence of common geriatric syndromes. On average, the patients of specialized geriatric hospital wards in our analysis lived in an own apartment or house prior to hospital admission, had 4 compensated and 6 uncompensated problems, a BI of 40 pts. on admission with a median increase of 15 points during hospital stay and a median length of stay of 16 days in the geriatric hospital department. However, the high number and the great variability of problems per person in this analysis documents the complexity of health care needs of geriatric patients. Uncompensated problems are generally addressed during specialized geriatric hospital treatment, but compensated problems may also have an impact on the complexity of the hospital treatment and the effort necessary for the multi-professional geriatric team. Therefore, we sought to combine both factors with distinct grading (compensated problems: 1 pt., uncompensated problems: 2 pts.). This calculated SEISAR symptom burden score could possibly serve as a distinctive feature to identify patients in need of geriatric health care.

However, as long as we do not have comparative data from older patients of non-geriatric hospital departments, it cannot be decided if SEISAR is appropriate to differentiate between patients with and without need of specialized geriatric hospital care. Nevertheless, the results give a good overview of the dominating functional and health problems of patients in acute care geriatric hospital units in Germany and the differentiation between compensated and uncompensated problems seems reasonable to characterize the requirements and guide the treatment focus towards individual unmet medical and geriatric needs. However, despite a detailed manual with many explanations and examples for the classification of problems, that was provided to all participating centers, it sometimes remained unclear when to classify a problem as uncompensated. As an example, currently it is difficult to exactly determine, when.

polypharmacy may be a compensated problem. Is it, compensated if, according to the manual, a patient is capable to deal with it or if there is a competent person responsible for the medication management? Or is polypharmacy compensated if a potentially inappropriate medication is missing or the medication has been reviewed by a geriatrician? The way the provided manual addresses these questions is fully appropriate for the original task of SEISAR in the emergency department. However, if SEISAR should be used to stratify the need of admission to an acute care geriatric hospital department in the future, such inaccuracies should be solved. Overall, all participating physicians judged the SEISAR as easy and fast to complete, if the patients had been medically examined by them before the completion. Complaints about uncertainties regarding problem classification were rarely expressed.

This study has several limitations. First, the data were obtained by the attending physician and not by trained study personnel. This may have led to underreporting of problems but on the other hand reflects how the SEISAR would be applied in clinical routine. Second and unfortunately, the study did not record age or gender of the participants. Therefore, the data could not be analyzed for age or gender effects. In previous studies in German geriatric wards, the mean age of patients was about 83 years with 63–70% being female [10–13]. Third, the change in status from uncompensated to compensated problems due to geriatric interventions during hospital stay was not recorded. Fourth, the comparison with older patients admitted to non-geriatric hospital departments is missing, both of which should be performed in future studies.

## Conclusion

SEISAR is an interesting standardized brief comprehensive geriatric assessment tool for the identification of compensated and uncompensated health problems in older persons. While designed to assure safe discharge of older patients from the emergency department, the use of SEISAR in specialized geriatric hospital units in this study characterized this population and highlighted the number, variability, and complexity of geriatric problems. However, the objectivity, validity and reliability in this setting has to be proven.

### Supplementary Information

**Additional file 1.** Prevalence of problems by change in barthel-index.

## Data Availability

The datasets used and/or analysed during the current study are available from the corresponding author on reasonable request.

## Abbreviations

BI: Barthel-Index
IQR: Interquartile ranges
ISAR: Identification of Seniors at Risk-score
max: maximum
min: minimum
SD: Standard deviations
