# Supplementary material for: Characterization of patients admitted to specialized geriatric acute care hospital units with the German version of the Standardized Evaluation and Intervention for Seniors at Risk (SEISAR) screening-instrument: a cross-sectional study
Source: BMC Geriatr. 2023 Sep 30;23:613. doi: 10.1186/s12877-023-04338-7 (PMC10542688; doi:10.1186/s12877-023-04338-7)
Supplement: Supplementary file 1 — Additional file 1. Prevalence of problems by change in barthel-index. [file 12877_2023_4338_MOESM1_ESM.doc]

**Additional file 1:** Prevalence of problems by change in barthel-index.

|  | barthel-index (n=26 missing) | |
| --- | --- | --- |
|  | no change/decline (n=131) | Increase (≥5)  (n=599) |
| **Communication:** |  |  |
| Impaired vision* |  |  |
| absent | 42 (32.1) | 213 (35.6) |
| compensated | 71 (54.2) | 320 (53.4) |
| uncompensated | 18 (13.7) | 55 (9.2) |
| Impaired hearing* |  |  |
| absent | 58 (44.3) | 303 (50.6) |
| compensated | 49 (37.4) | 206 (34.4) |
| uncompensated | 22 (16.8) | 74 (12.4) |
| **Cognition:** |  |  |
| Acute confusion/disorientation* |  |  |
| absent | 66 (50.4) | 413 (68.9) |
| compensated | 11 (8.4) | 45 (7.5) |
| **uncompensated** | **52 (39.7)** | **138 (23.0)** |
| Undiagnosed cognitive problems* |  |  |
| absent | 80 (61.1) | 476 (79.5) |
| compensated | 12 (9.2) | 31 (5.2) |
| **uncompensated** | **38 (29.0)** | **83 (13.9)** |
| **Nutrition:** |  |  |
| Recent weight loss/malnutrition* |  |  |
| absent | 60 (45.8) | 355 (59.3) |
| compensated | 14 (10.7) | 42 (7.0) |
| uncompensated | 57 (43.5) | 201 (33.6) |
| Substance abuse* |  |  |
| absent | 120 (91.6) | 564 (94.2) |
| compensated | 2 (1.5) | 6 (1.0) |
| uncompensated | 7 (5.3) | 23 (3.8) |
| **Mobility:** |  |  |
| Falls* |  | ) |
| absent | 42 (32.1) | 172 (28.7) |
| compensated | 7 (5.3) | 71 (11.9) |
| uncompensated | 82 (62.6) | 353 (58.9) |
| Problems walking/difficulty in using walking aid* |  |  |
| absent | 20 (15.3) | 143 (23.9) |
| compensated | 27 (20.3) | 180 (30.1) |
| **uncompensated** | **83 (63.4)** | **270 (45.1)** |
| **Activities of daily living:** |  |  |
| Difficulties with meal preparation* |  |  |
| absent | 32 (24.4) | 262 (43.7) |
| compensated | 52 (39.7) | 176 (29.4) |
| uncompensated | 46 (35.1) | 156 (26.0) |
| Limitations with basic hygiene* |  |  |
| absent | 29 (22.1) | 258 (43.1) |
| compensated | 50 (38.2) | 184 (30.7) |
| uncompensated | 50 (38.2) | 148 (24.7) |
| Incontinence* |  |  |
| absent | 50 (38.2) | 339 (56.6) |
| compensated | 41 (31.3) | 135 (22.5) |
| uncompensated | 38 (29.0) | 119 (19.9) |
| **Medication:** |  |  |
| Polypharmacy/new medication* |  |  |
| absent | 20 (15.3) | 86 (14.4) |
| compensated | 16 (12.2) | 71 (11.9) |
| uncompensated | 95 (72.5) | 437 (73.0) |
| Prescription management difficulties* |  |  |
| absent | 65 (49.6) | 375 (62.6) |
| compensated | 32 (24.4) | 112 (18.7) |
| uncompensated | 30 (22.9) | 101 (16.9) |
| **Behavior/affect:** |  |  |
| Depression* |  |  |
| absent | 99 (75.6) | 506 (84.5) |
| compensated | 7 (5.3) | 35 (5.8) |
| uncompensated | 24 (18.3) | 56 (9.3) |
| Agitation* |  |  |
| absent | 109 (83.2) | 556 (92.8) |
| compensated | 6 (4.6) | 14 (2.3) |
| uncompensated | 14 (10.7) | 24 (4.0) |
| **Active medical issues:** |  |  |
| Persistent presenting symptoms* |  |  |
| absent | 23 (17.6) | 165 (27.5) |
| compensated | 25 (19.1) | 141 (23.5) |
| uncompensated | 82 (62.6) | 289 (48.2) |
| Active co-morbidities* |  |  |
| absent | 31 (23.7) | 199 (33.2) |
| compensated | 21 (16.0) | 142 (23.7) |
| **uncompensated** | **77 (58.8)** | **253 (42.2)** |
| **Pain management:** |  |  |
| Persistent pain* |  |  |
| absent | 60 (45.8) | 271 (45.2) |
| compensated | 26 (19.8) | 153 (25.5) |
| uncompensated | 45 (34.4) | 173 (28.9) |
| Joint/bone pain* |  |  |
| absent | 65 (49.6) | 287 (47.9) |
| compensated | 27 (20.6) | 144 (24.0) |
| uncompensated | 39 (29.8) | 164 (27.4) |
| **Social:** |  |  |
| Insufficient support, lives alone* |  |  |
| absent | 68 (51.9) | 248 (41.4) |
| compensated | 36 (27.5) | 172 (28.7) |
| uncompensated | 24 (18.3) | 172 (28.7) |
| Social isolation/neglect* |  |  |
| absent | 99 (75.6) | 448 (74.8) |
| compensated | 24 (18.3) | 99 (16.5) |
| uncompensated | 7 (5.3) | 40 (6.7) |
| Previously refused service* |  |  |
| absent | 107 (84.8) | 508 (84.8) |
| compensated | 13 (8.7) | 52 (8.7) |
| uncompensated | 8 (6.1) | 27 (4.5) |

*missing values in up to 2.7% of cases per group

Significant differences (chi square test) are marked in bold.

;
